# Unilateral simultaneous optic nerve and choroidal infiltration – unusual presentation of metastatic disease in breast carcinoma

**DOI:** 10.3205/oc000187

**Published:** 2021-09-21

**Authors:** Poninder Kumar, Ashok Kumar, Srujana Dubakka, Jaya Kaushik, Mohini Agrawal

**Affiliations:** 1 Department of Ophthalmology, Armed Forces Medical College, Pune, India

## Abstract

Breast carcinoma metastasis can involve any ocular structures, but involvement of the optic nerve is extremely rare. Choroidal metastasis is usually multifocal as well as bilateral and occurs late. We report an unusual initial presentation of metastasis from breast cancer; unilateral infiltrative optic neuropathy with concurrent choroid metastatic deposits in an adequately treated middle-aged female. Our present case, wherein for the first time in the literature, we illustrated unilateral infiltrative optic neuropathy and choroidal metastatic deposits secondary to breast carcinoma, will increase our knowledge about the various potential ocular presentations of this relatively common malignant disease.

## Case description

A 41-year-old woman with a history of invasive ductal type of adenocarcinoma of her right breast presented with diminution of vision in the right eye of 15 days duration. The diagnosis of her right-sided stage IIIc breast carcinoma was made 6 years ago when she developed a lump and pain in the right side of her chest. She subsequently underwent a right-sided modified radical mastectomy followed by eight cycles of chemotherapy and external beam radiation of the thoracic wall. She remained stable thereafter and presented with diminution of vision in the right eye, six years after initial diagnosis of breast cancer.

**Figure 1:**
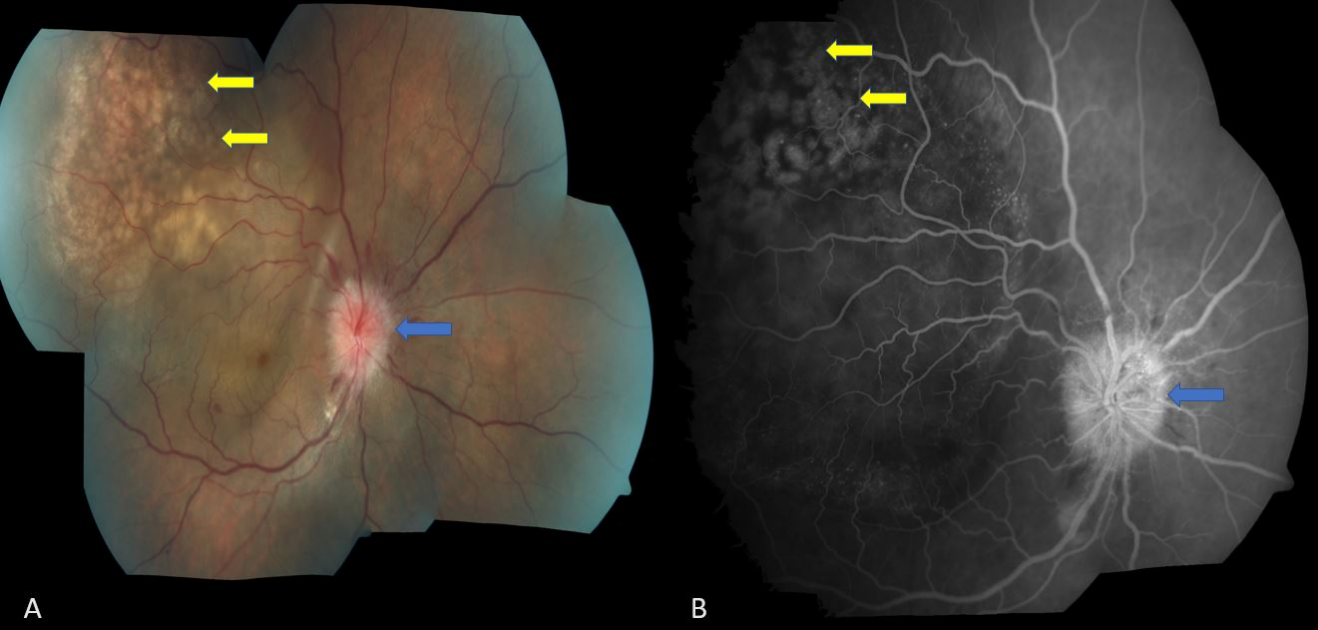
A) Fundus examination of the right eye showing diffuse enlargement of the optic disc, disc oedema with splinter hemorrhages in the surrounding retina (blue arrow) suggestive of optic nerve infiltration. There are multiple, homogenous, creamy yellow lesions with interspersed alterations of the retinal pigment epithelium along the supero-temporal vascular arcade (yellow arrows) associated with serous detachments of the fovea characteristic of choroidal metastatic deposits. B) Fundus fluorescein angiography of the same eye showing small hyperfluroscent lesion on the disc with no leakage (blue arrow) suggestive of optic nerve infiltration and pin-point hyperfluorescence (yellow arrows) in the area of choroidal deposits with dye pooling under the area of neurosensory detachment typical of choroidal metastasis.

On evaluation, the best corrected visual acuity (BCVA) was 3/60 in the right eye and 6/6 in the left eye. Examination of the right eye was completely unremarkable except for the presence of a relative afferent pupillary defect (RAPD). There was also the presence of trace vitreous cells on the anterior vitreous surface in the right eye. Fundus examination of the right eye revealed a diffuse enlargement of the optic disc, and disc oedema with splinter haemorrhages in the surrounding retina (blue arrow) suggestive of optic nerve infiltration. There were multiple, homogenous, creamy yellow lesions with interspersed alterations of the retinal pigment epithelium along the supero-temporal vascular arcade (yellow arrows) associated with serous detachments of the fovea characteristic of choroidal metastatic deposits (Figure 1A (Fig. 1)). Fundus fluorescein angiography showed a small hyperfluorescent lesion on the disc with no leakage (blue arrow) suggestive of optic nerve infiltration and pin-point hyperfluorescence (yellow arrows) in the area of choroidal deposits, and pooling of dye under the area of neurosensory detachment in the macular area typical of choroidal metastasis (Figure 1B (Fig. 1)). The contrast-enhanced magnetic resonance imaging (CE-MRI) confirmed infiltration of the intra-orbital part of the optic nerve in the right eye and choroidal deposits with exudative detachment of the retina.

In view of the patient’s present condition, her past history of breast cancer, optic nerve head infiltrative and characteristic choroidal metastatic features on fundus examination, and imaging findings, the diagnosis of infiltrative optic neuropathy with concurrent choroidal metastasis of the right eye secondary to breast carcinoma was made. The patient was referred to an oncologist to rule out other system involvement and necessary interventions.

## Discussion

Metastatic tumor is the most common ocular malignancy, and uveal tissue is the most favored site where cancer metastases develop [1]. The overall incidence of ocular breast cancer metastatic disease varies between 5%–30%, most commonly in the highly vascular choroid, followed by anterior segment and orbital structures, but rarely involves the optic nerve [2], [3]. Ocular metastasis from breast cancer is usually late. In most cases, lungs involvement occurs quite early.

Optic nerve head involvement in metastatic disease of the breast is seen in less than 5% of all intraocular metastases; appearing as a diffuse enlargement of the optic disc in about 84% of cases with a degree of secondary disc oedema and splinter haemorrhages [4]. In the present case, metastatic disease of the breast presented as unilateral optic nerve infiltration with localized choroidal metastasis away from the optic disc in one eye, manifesting with diminution of vision in the same eye as a first sign of metastasis. The visual acuity of our patient had diminished progressively, with impaired color vision due to an infiltrative optic neuropathy and exudative detachment associated with choroidal metastasis. She had a relative afferent pupillary defect due to unilateral involvement of the optic nerves of the right eye.

Optic disc infiltration is known to occur either due to direct extension of a choroidal tumor which is located close to the optic disc, or due to a spread of neoplastic cells to the circulation of the optic nerve head by blood route [5]. The hematogenous spread seems to be more plausible in our case, as the patient had unilateral optic nerve involvement with choroidal metastatic deposits located away from the optic disc. The patient was immediately referred to an oncologist for further evaluation and treatment.

## Conclusion

To conclude, breast carcinoma is a relatively common source of ocular metastasis and can have varied presentations depending on the site of metastasis. Unilateral infiltrative optic neuropathy with concurrent choroidal deposits as initial manifestation needs to be considered as differential diagnosis and potential cause of severe diminution of vision due to metastasis in patients of breast carcinoma.

## Notes

### Competing interests

The authors declare that they have no competing interests.
